# Anthocyanins as Promising Molecules Affecting Energy Homeostasis, Inflammation, and Gut Microbiota in Type 2 Diabetes with Special Reference to Impact of Acylation

**DOI:** 10.1021/acs.jafc.2c05879

**Published:** 2022-12-14

**Authors:** Kang Chen, Maaria Katariina Kortesniemi, Kaisa Marjut Linderborg, Baoru Yang

**Affiliations:** Food Sciences, Department of Life Technologies, University of Turku, FI-20014 Turku, Finland

**Keywords:** acylated anthocyanins, diabetes, energy metabolism, gut microbiota, inflammation

## Abstract

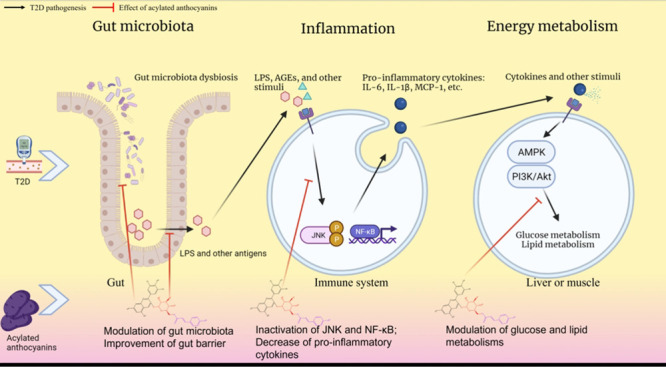

Anthocyanins, the red-orange to blue-violet colorants present in fruits, vegetables, and tubers, have antidiabetic properties expressed via modulating energy metabolism, inflammation, and gut microbiota. Acylation of the glycosyl moieties of anthocyanins alters the physicochemical properties of anthocyanins and improves their stability. Thus, acylated anthocyanins with probiotic-like property and lower bioavailability are likely to have different biological effects from nonacylated anthocyanins on diabetes. This work highlights recent findings on the antidiabetic effects of acylated anthocyanins from the perspectives of energy metabolism, inflammation, and gut microbiota compared to the nonacylated anthocyanins and particularly emphasizes the cellular and molecular mechanisms associated with the beneficial effects of these bioactive molecules, providing a new perspective to explore the different biological effects induced by structurally different anthocyanins. Acylated anthocyanins may have greater modulating effects on energy metabolism, inflammation, and gut microbiota in type 2 diabetes compared to nonacylated anthocyanins.

## 1 Introduction

Type 2 diabetes (T2D) is a disorder characterized by chronic hyperglycemia that results from impairments in insulin resistance and/or secretion. In T2D, impaired insulin function increases plasma glucose, nonesterified fatty acids, and branched-chain amino acids, which causes the defected energy metabolism and shifts energy metabolism from carbohydrate catabolism to fatty acid oxidation due to impaired insulin-stimulated glucose disposal.^1^ Insulin resistance is often accompanied by low-grade and chronic inflammation.^2^ In T2D, high-calorie intake, such as a high-fat diet, can trigger dysbiosis of gut microbiota and damage in the gut barrier, and subsequently induced endotoxemia, further aggravating insulin resistance and inflammation.^3^ Besides genetic predisposition, also an unhealthy lifestyle can contribute to the development of T2D.^4^ Healthy food can be a choice for the prevention and management of T2D.^4^

Anthocyanins, as a class of polyphenols giving red-orange to blue-violet colors in plants, have antioxidant and anti-inflammatory properties and can also positively affect energy homeostasis and gut health.^5^ These properties originate from radical scavenging properties, inhibition of carbohydrate digestion enzymes, and complex anthocyanin-gut microbiota interactions.^5^

Acylation of the sugar moiety of anthocyanins alters their physicochemical properties. Compared to nonacylated anthocyanins, acylated anthocyanins are more stable. For example, acylated anthocyanins have shown more resistance to changes in heat, pH, and light, stronger potential to inhibit lipid peroxidation, and higher antioxidant activity.^6^ Berries are a major dietary source of nonacylated anthocyanins,^7^ while acylated anthocyanins are naturally present in dark-colored vegetables and tubers. Earlier reviews have concluded that anthocyanins modulate energy homeostasis, inflammation, and gut microbiota in T2D.^4,8−15^ However, these reviews have only focused on the nonacylated anthocyanins. Although there are also reviews comparing the postprandial carbohydrate metabolism between acylated and nonacylated anthocyanins,^16^ there is limited information on how structurally different anthocyanins vary in their antidiabetic effects. This review summarizes the effects of acylated anthocyanins on energy homeostasis, inflammation, and gut microbiota in T2D and discusses the differences in the antidiabetic metabolism between acylated and nonacylated anthocyanins.

## 2 Nonacylated vs Acylated Anthocyanins: Structure, Stability, and Bioavailability

**Figure 1:**
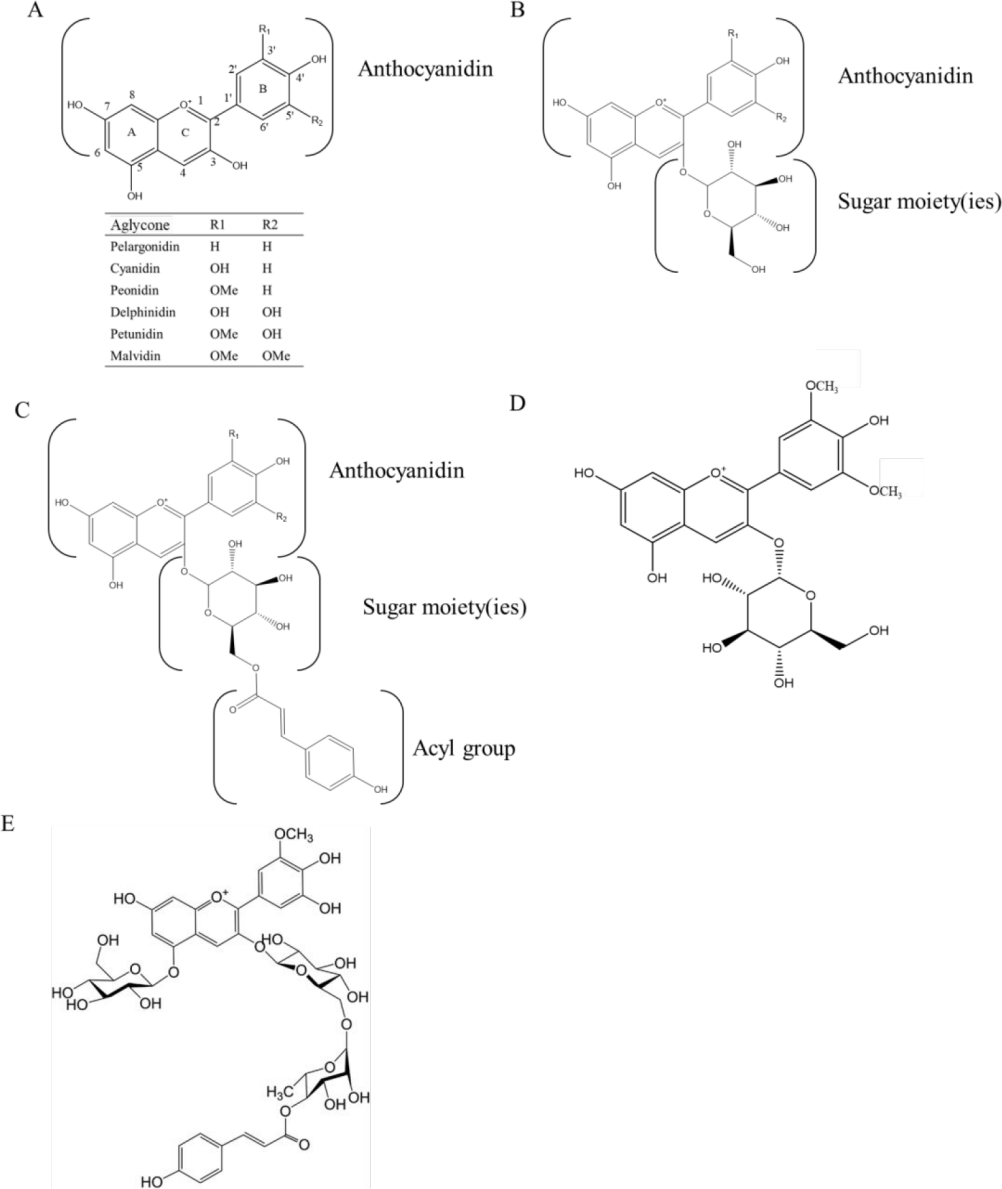
Structures of common anthocyanins. An example of nonacylated and acylated anthocyanins. Glucose is attached to the C3 position of anthocyanidin (A) to form nonacylated anthocyanin (B). Based on nonacylated anthocyanin, *p*-coumaric acid is acylated with a glucose residue at the C6 position to form acylated anthocyanin (C). Malvidin-3-*O*-glucoside (D). Petunidin-3-*O*-[6-*O*-(4-*O*-*E*-*p*-coumaroyl-*O*-α-l-rhamnopyranosyl)-β-d-glucopyranoside]-5-*O*-β-d-glucopyranoside (E), adapted with permission from ref (105). Copyright 2003 Elsevier.

Hundreds of different anthocyanins are known to occur naturally. Their structural diversity arises from the number and the position of the sugar moieties in the aglycones and acyl groups in the sugar moieties and the hydroxylation of aglycones. Cyanidin, petunidin, pelargonidin, delphinidin, peonidin, and malvidin are the six most common aglycones (anthocyanidins) (Figure 1A).

The sugar moieties attached to aglycones of anthocyanin can be acylated with organic acids by plant enzymes (Figure 1B,C). Examples of specific nonacylated anthocyanins in bilberry (*Vaccinium myrtillus*) (malvidin-3-*O*-glucoside) and acylated anthocyanins (petunidin-3-*O*-[6-*O*-(4-*O*-*E*-*p*-coumaroyl-*O*-α-l-rhamnopyranosyl)-β-d-glucopyranoside]-5-*O*-β-d-glucopyranoside) in purple potato are shown in Figure 1D,E, respectively. Common acyl groups include hydroxycinnamic acids and aliphatic acids. Multiple acyl groups can be found in the same molecules, such as in poly acylated anthocyanins. Acylated anthocyanins are abundant in purple-fleshed potato (*Solanum tuberosum*), purple sweet potato (*Ipomoea batatas*), red radish (*Raphanus sativus*), purple carrot (*Daucus carota*), and red cabbage (*Brassica oleracea*) as reviewed elsewhere.^16^

Acylation of plant secondary metabolites plays an important role in their physicochemical properties and biological activity.^17^ In plants, anthocyanin acyltransferases (ACT) are responsible for the acylation process of anthocyanins.^17^ The two main types of ACTs have been classified based on the acyl group donors: acyl-activated sugar and acyl-CoA as acyl group donors.^17^

The polarity of anthocyanins is decreased by glycosyl acylation, and the vulnerability of the flavylium cation to a nucleophilic attack by water is also decreased by stacking the acyl groups with the pyrylium ring.^16^ The hydrophilic initiation of the interaction between anthocyanins and their membrane carriers (bilitranslocase) during absorption is inhibited by the steric hindrance effect of acylated anthocyanins.^18^

The conjugated carbon–carbon double bonds in aromatic acyl groups can donate electrons and absorb light energy, which contribute to the stability of anthocyanins under light irradiation.^19^ Acylation of anthocyanin can substantially improve the resistance of acylated anthocyanins to a variety of physicochemical and biochemical factors (e.g., pH, heat, light, oxidation, and gastrointestinal digestion).^20^ Anthocyanins in colored plant organs can be used as alternatives for synthetic colorants, in which acylated anthocyanins primarily contribute the stable colorations.^21^ The acylated anthocyanins from red cabbage have shown more stability to heating at 80 °C and changes in pH than the nonacylated anthocyanins in grape skin, black currant, and elderberry extracts.^22^ Acylated anthocyanins have shown to be more stable under direct exposure to sunlight than the nonacylated anthocyanins in fruit juices,^23^ and the acylated anthocyanins synthesized by lipase-catalyzed transesterification have also shown to be more stable under illumination with white fluorescent light compared to the corresponding nonacylated glucosides.^24^ A recent study has shown that enzymatic acylation of delphinidin-3-*O*-glucoside, delphinidin-3-*O*-rutinoside, cyanidin-3-*O*-glucoside, and cyanidin-3-*O*-rutinoside with lauric acid (12:0) significantly improved the anthocyanins’ thermostability, antioxidant activity, and capacity to inhibit lipid peroxidation.^25^ Acylated anthocyanins from purple yam (*Dioscorea alata*) have exhibited a higher level of antioxidant activity than the corresponding nonacylated anthocyanins from the same source, for example, acylated anthocyanin cyanidin-3-*O*-(6-*O*-(6-*O*-(*E*)-sinapoyl-β-d-glucopyranosyl)-β-d-glucopyranosyl)-7-*O*-(6-*O*-(*E*)-sinapoyl-β-d-glucopyranosyl)-2′-*O*-(β-d-glucopyranosyl) has shown five times higher antioxidant activity compared to nonacylated anthocyanin cyanidin-3-*O*-(6-*O*-β-d-glucopyranosyl)-β-d-glucopyranosyl) *in vitro*.^6^

When discussing the biological impacts of anthocyanins, it is important to address their bioavailability.^16^ Dietary anthocyanins are metabolized by phase I and phase II enzymes to produce phenolic acids and their conjugates after being absorbed into enterocytes as intact glycosides or aglycones.^26^ Up to 65% of dietary anthocyanins are not absorbed in the stomach and upper intestine,^1^ so they pass through to the colon and are degraded extensively by gut microorganisms. This process shapes the gut microbiota profile and gut metabolic profile.^27^

As systematically reviewed by Jokioja et al.,^16^ acylated anthocyanins have lower transport efficiency, higher resistance to digestion, and higher susceptibility to fermentation by gut microbiota than nonacylated anthocyanins. In a bioavailability study, acylated anthocyanins from purple-fleshed sweet potato have shown lower bioavailability compared to nonacylated anthocyanins from red wine under simulated digestion conditions *in vitro*, with a degradation percentage of about 30% and 45% at the intestinal level, respectively.^28^ A clinical bioavailability study has shown an 11–14-fold lower recovery of acylated anthocyanins in urine and an 8–14-fold lower concentration in plasma compared to their nonacylated forms, using purple carrots as the source of nonacylated and acylated anthocyanins.^29^ A similar finding of acylated anthocyanins having lower transport efficiency than nonacylated anthocyanins has also been observed in a transepithelial transport experiment based on the intestinal Caco-2 cell line model.^30^ In addition, a recent research study has shown that the absorption efficiency (in gastric epithelial cells) of two acylated anthocyanins (peonidin-3-(6′-hydroxybenzoyl)-sophoroside-5-glucoside and peonidin-3-(6′-hydroxybenzoyl-6″-caffeoyl)-sophoroside-5-glucoside) purified from purple sweet potato was 20–30% lower than nonacylated malvidin-3-*O*-glucoside.^31^

Lower absorption efficiency of acylated and nonacylated anthocyanins has been seen when the glucose transporters GLUT1 and GLUT3 were blocked, suggesting that GLUT1 and GLUT3 were involved in anthocyanin absorption.^31^ A computational study comparing the affinity of acylated anthocyanin malvidin 3-*O*-(6-*O*-coumaroyl)-glucoside-5-*O*-glucoside with nonacylated anthocyanin malvidin 3,5-*O*-diglucoside to GLUT1 and GLUT3 showed acylated anthocyanin had stronger affinity to GLUT3 and the nonacylated anthocyanin had stronger affinity to GLUT1.^32^

Acylated anthocyanins may be more readily degraded by the gut microbiota in the gut. The fecal total anthocyanin content has been shown to increase by more than 10-fold after antibiotic treatment to knock down the gut microbiota in obese rats fed with a Concord grape supplement rich in acylated anthocyanins for 8 weeks.^33^ This change was greater than that observed in obese rats fed with an equivalent dose of nonacylated anthocyanins in berries.^33^ Acylated anthocyanin cyanidin-3-(6′′-malonyl)-glucoside is likely to be more readily available for degradation by gut microbiota than its nonacylated form since the ratio between these two anthocyanins in the cecal contents in rats has been observed to shift to favor the nonacylated anthocyanin when compared to the ratio in the original food, red orange juice.^34^

## 3 Nonacylated vs Acylated Anthocyanins: Effects on Type 2 Diabetes

The antidiabetic properties of anthocyanins in nonacylated form have been reviewed extensively.^4,8,9,11,12,15,27^ The effects on energy metabolism, inflammation, and gut microbiota include (1) inhibition of digestive enzymes; (2) modulation of adenosine monophosphate-activated protein kinase (AMPK) activation and AMPK-mediated GLUT4 expression and translocation; (3) suppression of peroxisome proliferator-activated receptor gamma (PPARγ) and activation of phosphoinositide 3 kinase/protein kinase B (PI3K/AKT)-mediated energy metabolism; (4) suppression of nuclear factor κB (NF-κB) activation and downstream of inflammatory cytokines expression such as interleukins IL-1β and IL-6; and (5) activation of nuclear factor erythroid 2-related factor 2 (Nrf2). Recent studies on the antidiabetic effects of acylated anthocyanins and nonacylated anthocyanins are summarized in Tables 1 and 2 and reviewed in detail in the subsequent sections.

**Table 1:** An Overview of Studies Involved in Antidiabetic Effects of Nonacylated Anthocyanins^a^^,^^b^

Source of anthocyanins	Main anthocyanin(s)	Model	Effects
Pure nonacylated anthocyanins^106,107^	18–20 varieties of nonacylated anthocyanins	Enzyme inhibition study	Inhibited α-amylase and α-glucosidase. Structure-dependent enzymes inhibition property was observed
Purified nonacylated anthocyanin^87^	Cya-3-glc	3T3-L1 adipocytes and human omental adipocytes	Enhanced glucose transport, GLUT4 membrane translocation, and insulin sensitivity
Purified anthocyanin^59^	Cya-3-glc	KKA^y^ diabetic mice; Diet containing 100 mg/kg; 12 weeks	Ameliorated hepatic steatosis by decreasing hepatic mtGPAT1 activity. Anthocyanin inhibited high glucose-induced hepatic mtGPAT1 activation and prevents fatty acid synthesis through protein kinase C
Purified anthocyanin^108^	Cya-3-glc	*db/db* diabetic mice; Diet containing 100 mg/kg; 8 weeks	Increased the GSH synthesis through protein kinase A/cAMP-response element binding protein-dependent induction of Gclc expression
			Attenuated lipid peroxidation, neutrophil infiltration, and hepatic steatosis
Purified anthocyanins from Cornus fruits (*C. officinalis* and *C. mas*)^109^	Cya-3-glc, del-3-glc, cya-3-gal, and pg-3-gal, and anthocyanidins	Rodent pancreatic β-cells (INS-1832/13)	Enhanced insulin secretion and prevent insulin resistance
Anthocyanins extract from mulberry (*Morus alba*)^44^	Cya-3-glc, cya-3-rut, pg-3-glc	*db/db* mice; 50 and 125 mg/kg body weight; 8 weeks	Modulated AKT, GSK3β and FOXO1 in liver, muscle, and adipose tissues. Decreased triglycerides, LDL, insulin, blood glucose, leptin, β cell protection. Reversed insulin resistance by regulating AMPK/ACC/mTOR pathway
Anthocyanin extract from mulberry (*Morus alba*) fruits^110^	Que-3-glc and other phenolic acids	HFD- induced obese Syrian golden hamster; Diet containing 2% (w/w); 12 weeks	Reduced serum triacylglycerol, cholesterol, free fatty acid, and the LDL/HDL ratio
			Decreased hepatic lipids and hepatic PPAR-γ, fatty acid synthase, and HMG-CoA reductase
Anthocyanins extract from mulberry (*Morus alba*)^52^	Cya-3-glc, cya-3-rut	*db/db* diabetic mice; diet containing 0.5%, w/w; 6 weeks	Decreased blood levels of glucose and HbA1c
			Increased insulin sensitivity
			Activated pAMPK and p-AKT substrate of 160 kDa (pAS160) and enhanced GLUT4 in skeletal muscles
			Increased pAMPK and decreased the levels of G6pase and PEPCK in the liver
Anthocyanins extract from mulberry (*Morus alba*)^111^	Cya-3-glc, cya-3-rut, pg-3-glc, pg-3-rut	Zucker diabetic fatty rats; 125 and 250 mg/kg body weight; 5 weeks	Decreased glucose level
			Maintained insulin level and β cell histology
Anthocyanin extract from mulberry (*Morus Australis* Poir)^112^	Cya-3-glc, cya-3-rut, pg-3-glc	HFD-induced obese C57b1/6J mice; Diet containing 4% (w/w); 90 days	Decreased body weight, food intake, cholesterol, triglycerides, glucose, and leptin
Anthocyanin extrat from bilberry (*Vaccinium myrtillus*)^113^	Del-, cya-, pet-, peo-, and mal- derived nonacylated anthocyanin	High-sucrose diet induced insulin-resistant mice; 0.2 mg/mL anthocyanin in drink water;15 weeks	Showed antioxidant property and changed genes expression in metabolic pathway (*ACC1, Bcl2, Akt, mTOR, GPDH1, HK2, GLUT1*, and *GLUT4*)
Commercial bilberry (*Vaccinium myrtillus*) anthocyanins extract capsule^114^	Del-3-gal, del-3-glc, del-3-ara, cya-3-gal, cya-3-glc	16 overweight volunteers; 0.47 g bilberry extract (36% (w/w) anthocyanins) for one dose	Improved oral glucose tolerance but not for plasma insulin level and anti-inflammatory markers
Commercial bilberry anthocyanin extract (*Vaccinium myrtillus*)^49^	Not available	KK-A^y^ diabetic mice; Diet containing 2.7% (w/w); 5 weeks	Decreased blood glucose and triglycerides and cholesterol. Improved insulin sensitivity
			Inactivated acetyl-CoA carboxylase and activated PPARa, acyl-CoA oxidase, and carnitine palmitoyltransferase-1A in liver
			Decreased PEPCK and G6pase in liver
			Activated AMPK in white adipose tissue, skeletal muscle, and the liver
			Upregulated GLUT4 in white adipose tissue and skeletal muscle
Freeze-dried highbush blueberries (50/50 blend of *Vaccinium virgatum* and *V. corymbosum*)^115^	Not available	52 men with T2D, 22 g freeze-dried blueberry twice a day for 8 weeks	Lowered hemoglobin A1c, triglycerides, AST, and ALT
			Fasting plasma glucose, serum insulin, total cholesterol, LDL, HDL, C-reactive protein concentrations, blood pressure, and body weight were not changed
Anthocyanins powder from two blueberries (Tifblue and Rubel cultivars, 1:1)^116^	Del-3-gal, peo-3-glc, mal-3-gal, cya-3-gal, pet-3-gal	Zucker diabetic fatty rats; Diet containing 2% (w/w); 90 days	Reduced triglycerides, fasting insulin. Improved insulin sensitivity
			Reduced abdominal fat mass, increased adipose and skeletal muscle PPAR activity, and affected PPAR transcripts involved in fat oxidation and glucose uptake/oxidation (*Fas, Irs1, Pfk, Glut4*)
Anthocyanins extract from honeyberry (*Lonicera caerulea*)^51^	Nonacylated anthocyanin dominant by cya-3-glc	HFD-induced obese ICR mice; diet containing 0.5%-1%, w/w; 6 weeks	Decreased the expressions of adipogenic genes (SREBP-1c, C/EBPα, PPARγ, FAS) in liver
			Increased mRNA and protein levels of CPT-1 and PPARα and ncreased the phosphorylation of AMPK and ACC in liver
Anthocyanin extract from purple corn (*Zea mays*)^46^	Nonacylated anthocyanins dominant by cya-3-glc	*db/db* diabetic mice; 10 or 50 mg/kg body weight; 8 weeks	Increased C-peptide and adiponectin and decreased HbA1c and glucose levels in plasma
			Prevented pancreatic β-cell damage and increased insulin content
			Increased the phosphorylation of AMPK and decreased PEPCK, G6pase genes in liver, and increased GLUT4 expression in skeletal muscle
Anthocyanin extracts from 20 purple maize genotypes^117^	Cya-3-glc, pg-3-glc, peo-3-glc and corresponding acylated forms	3T3-L1 adipocytes	Regulated TNF-α, IL-6 and MCP-1 and improved insulin sensitivity
Anthocyanin extract from purple rice (*Oryza sativa*)^71^	Cya-3-glc	STZ-induced diabetes SD rat; 250 mg/kg body weight; 4 weeks	Decreased blood glucose and gene expression of COX-2 and IL-6 inflammatory marker in heart
			Decreased TLR4 protein and p65-NF-κB levels in heart
			Activated p-Ikkα/βin heart

a Note: Anthocyanidins: cya, cyanidin; del, delphinidin; mv, malvidin; pg, pelargonidin; peo, peonidin; pet, petunidin. Sugar moieties: glc, glucopyranoside; gal, galactoside; rut, rutinoside; sam, sambubioside.

b Abbreviations: ACC, acetyl-CoA carboxylase; AKT, protein kinase B; AMPK, AMP-activating protein kinase; CPT, carnitine palmitoyltransferase; C/EBPα, CCAAT enhancer binding protein α; FAS, fatty acid synthase; FOXO1, forkhead box protein O1; Gclc, glutamate–cysteine ligase catalytic subunit; GSK3, Glycogen synthase kinase-3; GLUT, glucose transporter; G6pase, Glucose 6-phosphatase; HbA1c, hemoglobin A1c; HFD, high-fat diet; HMG-CoA, 3-hydroxy-3-methylglutaryl-CoA; IL, interleukin; IRS-1, Insulin receptor substrate 1; mtGPAT1, mitochondria glycerol-3-phosphate acyltransferase 1; mTOR, mammalian target of rapamycin; NF-κB, nuclear factor-κB; SREBP-2, sterol regulatory element-binding protein 2; TNF, tumor necrosis factor; PEPCK, Phosphoenolpyruvate carboxykinase; PPARγ, peroxisome proliferator-activated receptor γ; VCAM-1, vascular cell adhesion protein 1.

**Table 2:** An Overview of Studies Involved in Antidiabetic Effect of Acylated Anthocyanins and Comparison of Potential Antidiabetic Effects between Nonacylated and Acylated Anthocyanins^a^^,^^b^

Source of anthocyanins	Main anthocyanin(s)	Model	Effects
*The potential antidiabetic effects of acylated anthocyanins*			
Acylated anthocyanins extract from purple sweet potato (*Ipomoea batatas*)^79^	Cya-3-(6″-caf-6′′′-hba-sop)-5-glc, peo-3-(6″,6′′′-dicaf-sop)-5-glc, peo-3-(6″-caf-6′′′-p-hba-sop)-5-glc, peo-3-(6″-caf-6′′′-fer-sop)-5-glc	Enzyme inhibition study; human liver cell line HepG2 cells	Inhibited α-amylase, α-glucosidase, and xanthine oxidase; phenolic compound but not anthocyanin faction induced the transcription factor Nrf2 and Nrf2 target gene *Gclc*
Acylated anthocyanins extract from purple sweet potato (*Ipomoea batatas*)^118^	Not available	3T3-L1 adipocytes	Suppressed leptin secretion. Exerted anti-inflammatory, lipolytic effects on adipocytes and radical scavenging and reducing activity
Diacylated anthocyanin extracts from purple sweet potatoes (*Ipomoea batatas* L. cultivar Eshu No. 8)^39^	Peo-3-(6′-caf-6′′-hba-sop)-5-glc, peo-3-(6′-caffeoyl-6′′-fer-sop)-5-glc	SD rats. 80 and 160 mg/kg. One dose	Decreased postprandial blood glucose
Purple sweet potato (cultivar ‘*NingZi No. 2*’) anthocyanin extract^119^	Peo-3-caf-sop-5-glc, peo-3- (6′′,6′′′-dicaf-sop)-5-glc, peo-3-caf-hba-sop-5-glc, peo-3-(6′′-caf-6′′′-fer-sop)-5-glc, cya3-(6′′-caf-6′′′-fersop)-5-glc	HFD-indued obese SD rat; 100–400 mg/kg bodyweight; 6 weeks	Decreased adipocyte number and size of adipose tissue
			Decreased glucose, triglyceride, and total cholesterol levels
			Reduced the level of ROS and inhibited the receptor of AGE products and thioredoxin interacting protein in the hypothalamus
			Preserved the leptin signaling capability, decreased in hypothalamic AMPK activity
Anthocyanin extract from purple sweet potato (*Ipomoea batatas*)^74^	Peo-3-(6-caf-glc-β-glc)-5-glc, peo-3 -(2-(6-caf-glc)-6-caf-glc)-5-glc, peo-3-(2-(6-fer-glc)-6-caf-glcp)-5-glc, cya-3-(6-cou)-glc	HFD-induced obese C57BL/6 mice; 700 mg/kg/day; 20 weeks	Ameliorated obesity and liver injuries. Blocked hepatic oxidative stress. Restored NAD^+^ level in liver
			Suppressed the NF-κBp65 nuclear translocation, NOD1/2 signaling, the NLRP3 inflammasome activation and inflammation-related genes (*TNF-α*, *MCP-1*, and *IL-1*) in liver
Anthocyanin extract from purple sweet potato (*Ipomoea batatas*)^120^	Peo-3-(6-caf-glc-β-glc)-5-glc, peo-3 -(2-(6-caf-glc)-6-caf-glc)-5-glc, peo-3-(2-(6-fer-glc)-6-caf-glc)-5-glc, cya-3-(6-cou)-glc	HFD-induced obese C57BL/6 mice; 500 mg/kg/day; 32 weeks	Alleviated the cognitive impairment
			Decreased the expression of Iba1, TNF-α, IL-1β, SOCS3, galectin-3 in hippocampus
			Increased insulin signaling molecules including the p-IRS1 (Tyr608), PI3K p110α and p-AKT (Ser473)
			Increased Bcl-2 expression and diminished the Bak and the cleaved-caspase 3 expressions in hippocampus
Anthocyanin extract from purple sweet potato (*Ipomoea batatas*)^50^	Peo-3-(6-caf-glc-β-glc)-5-glc, peo-3 -(2-(6-caf-glc-6-caf-glc)-5-glc, peo-3-(2-(6-fer-glc)-6-caf-glc)-5-glc, cya-3-(6-cou)-glc	HFD-induced obese ICR mice; 200 mg/kg per day;4 weeks	Reduced weight gain and hepatic triglyceride accumulation and improved serum lipid parameters
			Increased the phosphorylation of AMPK and ACC in the liver
Anthocyanin extract from purple sweet potato (*Ipomoea batatas*)^45^	Cya- and peo-derived acylated anthocyanins	HFD-induced obese ICR mice; 700 mg/kg/day; 20 weeks	Improved the fasting blood glucose level, glucose, insulin tolerance and oxidative-stress-mediated endoplasmic reticulum stress
			Suppressed ROS production and GSH and antioxidant enzymes’ activities. Suppressed the JNK1and Ikb kinase β activation and NF-κB p65 nuclear translocation
			Restored the impairment of the insulin receptor substrate-1/phosphoinositide 3 kinase/protein kinase B (AKT) insulin signaling in the livers
Anthocyanin extract from purple sweet potato (*Ipomoea batatas*)^48^	Cya-3-sop-5-glc, peo-3-sop-5-glc, cya-3-hba-sop-5-glc, peo-3-hba-sop-5-glc, cya-3-(6″-fer- sop)-5-glc, peo-3-(6″-fer-sop)-5-glc, cya-3-caff-hba-sop-5-glc, cya-3-(6″-caff-sop)-5-glc, cya-3-(6″-caf-6″′-fer-sop)-5-glc, peo-3-caf-hba-sop-5-glc, peo-3-caf-sop-5-glc, peo-3-(6″-caf-6″′-fer-sop)-5-glc, Peo-3-(6″-hba-6″′-fer-sop)-5-glc	HFD and streptozotocin- induced obese ICR mice; 500 mg/kg body weight; 12 weeks	Activated AMPK in liver
			Increased GLUT4, glucokinase, and insulin receptor α in liver
			Upregulated glycolysis key genes (*Pfk* and *Pkm*)
			Downregulated gluconeogenic genes (*G6pase* and *Pepck*).
Anthocyanin extract from Blue Congo potatoes^121^	Acylated anthocyanins dominant by pet-3-cou-rut-5-glc	Streptozotocin- induced diabetic Wistar rats; 165 mg/kg bodyweight; 2 weeks	Lowered blood glucose, glycated hemoglobin, malondialdehyde levels in plasma
			Restored antioxidant enzyme activities
			Improved glucose tolerance
			Inhibited oxidative modified proteins OMP, AGE, and advanced oxidation protein products formation in plasma
Acylated anthocyanin extract from purple potato (*Solanum tuberosum* L. var. “Synkeä Sakari”)^122^	Acylated anthocyanins dominated by pet-cou-rut-glc and peo-cou-rut-glc	17 healthy volunteers; postprandial study; a meal containing acylated anthocyanins (152 mg)	Acylated anthocyanin extract alleviates postprandial glycemia and insulinemia and affects postprandial inflammation
Anthocyanin extract from black goji berry (*Lycium ruthenicum*)^75^	Pet-3-rut-cou-5-glc as the main anthocyanin	HFD and vitamin-D3- induced atherosclerosis rat, 50–200 mg/kg body weight; 8 weeks	Decreased total glyceride, total cholesterol, low density lipoprotein, TNF-α, IL-6 levels, and atherogenic index and increased HDL-C concentrations
			Upregulated NF-κB, VCAM-1, and CYP7A1, and downregulated SREBP-2
Anthocyanin extract from black goji berry (*Lycium ruthenicum*)^53^	Del-3–6″-rha-glc-5-glc, pet-3-rut-cou-5-glc, pet-3-rut-caf-5-glc, pet-3–6-cou-rha-pyr)-glc-5-glc	HFD-induced insulin resistance C57BL/6J mice; 50–200 mg/kg body weight; 12 weeks	Decreased the weight gain, hepatic lipid, dyslipidemia, inflammation, and oxidative stress
			Inactivated TLR4/NF-κB/JNK in the liver tissues and ameliorated oxidative stress and insulin resistance by activating the Nrf2/HO-1/NQO1 and IRS-1/AKT pathways
*Comparison of potential antidiabetic effects between nonacylated and acylated anthocyanins*			
Pure nonacylated anthocyanins and diacylated anthocyanins^38^	Cya-3-(2-(6-fer-glc)-6-caf-glc)-5-glc, cya and peo-3-(2-(6-fer-glc)-6-caf-glc)-5-glc, pg-3-(2-(6–3-glc-caf)-glc)-6-caf-glc)-5-glc, pg-3-(2-(6-caf-glc)-6-caf-glc)-5-glc, pg, cya, and peo-3-(2-glc-glc)-5-glc-3-sop-5-glc	Enzyme inhibition study	Diacylated anthocyanin showed the best ability to inhibit α-glucosidase
Nonacylated anthocyanin extracts from (*V. corymbosum* L. × *V. angustifolium* Aiton.; *V. ashei* Reade) berries; Monoacylated and diacylated extracts from purple sweet potatoes (*Ipomoea batatas* cultivar Eshu No. 8)^39^	Del-, cya-, pet-, peo-, and mal-3-gal, glc, ara; cy-, pet-, and peo-3-hba-sop-5-glc; cy and peo-3-(6′′-caf-sop)-5-glc; cya and peo-fer-sop-5-glc; cya-3-caf-sop-5-glc; peo-3-(6′-caf-6′′-hba-sop)-5-glc; peo-3-(6′-caffeoyl-6′′-fer-sop)-5-glc	Enzyme inhibition study	Diacylated anthocyanin extracts showed the highest inhibition ability of α-amylase and α-glucosidase than monoacylated anthocyanin extracts and deacylated anthocyanin extract
Acylated anthocyanin extract from purple carrot and pure cya-3-glc and del-3-rut^54^	Cya-3-(2″-xyl-6-glc-gal), cya-3-(2′′-xyl-gal), cya-3-(2′′-xylose-6′′-sin-glc-gal, cya-3-(2′′-xyl-6′′-fer-glc-gal, cya-3-(2′′-xyl-6′′(4-cou)-glc-gal	Wistar rats; Single intragastric doses	Acylated anthocyanin induced highest level of AKT phosphorylation in aorta than cya-3-glc and del-3-rut
Nonacylated anthocyanin extract from bilberry (*Vaccinium myrtillus*) and acylated anthocyanin extract from purple potato (*Solanum tuberosum* var. “Synkeä Sakari”)^58^	Nonacylated anthocyanins dominated by del-3-gal, del-3-glc, cya-3-gal, del-3-ara, cya-3-glc;	Zucker diabetic fatty rats; daily doses of 25–50 mg/kg body weight; 8 weeks	Both anthocyanin extracts decreased the levels of plasma glucose, branched-chain amino acids, and improved lipid profiles. Acylated anthocyanin extract increased the glutamine/glutamate ratio and decreased the levels of glycerol and metabolites involved in glycolysis. Acylated anthocyanin extract decreased the hepatic *TBC1D1* and *G6PC* mRNA levels
	Acylated anthocyanins dominated by pet-cou-rut-glc and peo-cou-rut-glc		
Nonacylated anthocyanin extract from bilberry (*Vaccinium myrtillus*) and acylated anthocyanin extract from purple potato (*Solanum tuberosum* var. “Synkeä Sakari”)^57^	Nonacylated anthocyanins dominated by del-3-gal, del-3-glc, cya-3-gal, del-3-ara, cya-3-glc;	Zucker diabetic fatty rats; daily doses of 25–50 mg/kg body weight; 8 weeks	Both anthocyanin extracts restored the levels of metabolites (glucose, lactate, alanine, and pyruvate) and expression of genes (*G6pac*, *Pck1*, *Pklr*, and *Gck*) involved in glycolysis and gluconeogenesis. Acylated anthocyanin extract decreased the hepatic glutamine level. Nonacylated anthocyanin extract regulated the expression of *Mgat4a*, *Gstm6*, and *Lpl*, whereas acylated anthocyanin extract modified the expression of *Mgat4a*, *Jun*, *Fos*, and *Egr1*
	Acylated anthocyanins dominated by pet-cou-rut-glc and peo-cou-rut-glc		

a Note: Anthocyanidins: cya, cyanidin; del, delphinidin; mv, malvidin; pg, pelargonidin; peo, peonidin; pet, petunidin. Acyl moieties: ace, acetyl; caf, caffeoyl; cou, coumaroyl; hba, hydroxybenzoyl; mal, malonyl; oxa, oxaloyl; sin, sinapoyl; suc, succinyl; pyr, pyranosyl. Sugar moieties: glc, glucopyranoside; gal, galactoside; rut, rutinoside; sop, sophoroside; xyl, xyloside.

b Abbreviations: ACC, acetyl-CoA carboxylase; AKT, protein kinase B; AMPK, AMP-activating protein kinase; Bcl-2, B-cell lymphoma 2; CPT, carnitine palmitoyltransferase; CYP7A1, cytochrome P450 family 7 subfamily A member 1; C/EBPα, CCAAT enhancer binding protein α; FAS, fatty acid synthase; FOXO1, forkhead box protein O1; Gclc, glutamate–cysteine ligase catalytic subunit; GSK3, Glycogen synthase kinase-3; GLUT, glucose transporter; G6pase, Glucose 6-phosphatase; HbA1c, hemoglobin A1c; HFD, high-fat diet; HMG-CoA, 3-hydroxy-3-methylglutaryl-CoA; HO-1, Heme oxygenase 1; IL, interleukin; IRS-1, Insulin receptor substrate 1; mtGPAT1, mitochondria glycerol-3-phosphate acyltransferase 1; mTOR, mammalian target of rapamycin; NF-κB, nuclear factor-κB; NLRP3, NOD-, LRR- and pyrin domain-containing protein 3; Nrf2, nuclear factor-erythroid factor 2-related factor 2; NOD, nucleotide-binding oligomerization domain; NQO1, NAD(P)H quinone dehydrogenase 1; SOCS3, suppressor of cytokine signaling 3; SREBP-2, sterol regulatory element-binding protein 2; TNF, tumor necrosis factor; PEPCK, Phosphoenolpyruvate carboxykinase; PPARγ, peroxisome proliferator-activated receptor γ; VCAM-1, vascular cell adhesion protein 1.

### 3.1 Inhibition of Carbohydrate Digestion Enzymes

Anthocyanins from plants are known to inhibit α-glucosidase and α-amylase, the key enzymes responsible for the digestion of dietary carbohydrates to glucose.^16^ Recent developments in structure-based design and computational techniques have shown that, out of 665 monomeric anthocyanins (nonacylated anthocyanins), pelargonidin-3-*O*-rutinoside and malvidin-3-*O*-arabinoside have the lowest binding energies with human pancreatic amylase and to interact with its amino acid residues (Asp197 and Glu233) through hydrogen bonds, thereby limiting its catalytic mechanism.^35^ Also, acylated anthocyanin extracts of purple potatoes and purple carrots have been reported to decrease the intestinal glucose uptake in a gastrointestinal model.^36^

A recent metabolomic study has shown cecal sugar levels (glucose, arabinose, galactose, xylose) were higher in rats fed with acylated anthocyanin extract (from purple potato, *S. tuberosum* var. “Synkeä Sakari”) and nonacylated anthocyanin extracts (from bilberry), with the acylated anthocyanin extract fed group showing the highest levels of those sugars, and the inhibition of digestive enzymes by anthocyanins might contribute to this result.^37^ Acylated anthocyanins have been observed to have more potent inhibitory effects on α-glucosidase and α-amylase than nonacylated ones.^38,39^ An *in vitro* study comparing the inhibitory effect of nonacylated anthocyanins fraction (547.94 ± 0.88 mg/g) from berries, monoacylated anthocyanins fraction (322.64 ± 4.84 mg/g) from purple sweet potatoes, and diacylated anthocyanins fraction (541.00 ± 7.06 mg/g) from purple sweet potatoes against α-glucosidase and α-amylase has shown that diacylated anthocyanins had the most potent inhibitory effect on these two enzymes.^39^ The inhibitory activity of the diacylated anthocyanins fraction on α-glucosidase is comparable to that of acarbose, one of the antidiabetic medications to decrease the digestion and absorption of carbohydrates.^39^ Feeding this diacylated anthocyanins fraction to SD rats has significantly decreased blood glucose level by 20.5% after a standard meal administration containing starch.^39^ The most potent inhibitory activity of diacylated anthocyanins to α-glucosidase might be due to the higher affinity to certain key enzyme binding sites.^39^ Moreover, protein-anthocyanin binding can prevent acylated anthocyanins from degrading, increasing their antioxidant activity.^40^ The inhibitory effect of monoacylated anthocyanins to α-glucosidase was, however, not significantly different from nonacylated anthocyanins, possibly because of a lower concentration.^39^

### 3.2 Modulation of Energy Metabolism

The energy metabolism is regulated by a number of kinases systems and related pathways, such as AMPK and PI3K/AKT pathways, which are the primary effectors in response to metabolic stress and crucial to energy metabolism and have been considered as therapeutic targets in metabolic syndrome, especially diabetes.^41^ PI3K/AKT, which controls energy metabolism and cell differentiation, proliferation, motility, and survival, can be activated by both exogenous and endogenous stimuli such as environmental stresses, insulin, growth hormones, and cytokines.^41,42^ Adipocytokines and several physiological factors, such as oxidative stress, hypoxia, glucose deprivation, and muscle contraction, can activate AMPK pathway.^41,43^

AKT and AMPK have been reported to activated by both types of anthocyanins in T2D (Tables 1 and 2). The PI3K/AKT pathway is necessary for insulin-dependent regulation of glucose and lipid metabolisms by regulating gluconeogenesis (FOXO1, Forkhead box protein O1), glycogen synthesis (GSK3β, glycogen synthase kinase 3β), and glucose uptake (TBC1D4, TBC1 domain family member 4).^41,42^ The AMPK pathway also participates in glucose and lipid metabolisms such as inhibiting glycogen synthesis (GYS1, muscle glycogen synthase) and lipogenesis (ACC, acetyl-CoA carboxylase α), activating glucose uptake (AS160, a substrate for the protein kinase AKT that links insulin signaling and GLUT4 trafficking), and enhancing β-oxidation (MCD, malonyl-CoA decarboxylase,).^41^

Since the liver and muscle are the main energy expenditure organs, the energy metabolism-regulatory effects of the two types of anthocyanins on the liver and muscle are presented in this review. Similar to nonacylated anthocyanins, we found feeding acylated anthocyanin extracts for 12–32 weeks (at daily doses of 200–700 mg/kg body weight) to streptozotocin- or high-fat diet-induced diabetic animal models have shown hypoglycemic effects by modulating hepatic AKT^44,45^ and hepatic AMPK.^46−49^ Activation of ACC mediated by AMPK contributing to lipid synthesis and suppression of *glucose 6*-*phosphatase (G6pase)* and phosphoenolpyruvate carboxykinase (PEPCK) mediated by PI3K/AKT leading to gluconeogenesis were observed by the intervention of nonacylated anthocyanin extract from berries or acylated anthocyanin extract from purple sweet potatoes (Tables 1 and 2).^46−49^ Mice fed with a high-fat diet and purple sweet potato acylated anthocyanin extract for 4 weeks at a daily dose of 200 mg/kg body weight have shown a decreased serum glucose by ca. 30% and induced hepatic phosphorylation of AMPK and ACC compared to the obese mice.^50^ Similarly, feeding mice with high-fat diet containing nonacylated anthocyanin extract from honeyberry (*Lonicera caerulea*) predominant in cyanidin-3-*O*-glucoside has induced hepatic phosphorylation of ACC and AMPK.^51^ The *db/db* diabetic mice fed with mulberry (*Morus alba*) nonacylated anthocyanin extract for 2 weeks had decreased blood glucose levels by ca. 35% and induced hepatic activation of AKT as well as upregulated *G6pase* and PEPCK levels.^52^ A study found that intervention of purple sweet potato acylated anthocyanin extract fed to high-fat diet-induced obese mice for 5 weeks has activated IRS-1 (Insulin receptor substrate 1)/PI3K/AKT pathway and contributed to the hypoglycemic effect (ca. 30% decrease in blood glucose).^45^ Feeding acylated anthocyanin extract from purple sweet potato for 4 weeks at a daily dose of 500 mg/kg body weight to diabetic mice induced by high-fat diet and STZ induced the AKT pathway and increased hepatic levels of PEPCK and G6pase, which led to ca. 35% blood glucose decrease in the treatment group.^48^ Acylated anthocyanins from black goji berry (*Lycium ruthenicum*) upregulated hepatic activation of AKT and gene expression of GLUT4, G6pase, and PEPCK in obese mice, which reversed glucose levels in normal healthy mice.^53^

Compared to the liver, the regulating influence of anthocyanins on energy metabolism in muscles has received less attention. Feeding a diet containing 2.7% (w/w) nonacylated anthocyanin extract from bilberry to KK-A^y^ diabetic mice for 5 weeks decreased blood glucose levels ca. 30% and activated AMPK in skeletal muscle, the liver, and adipose tissue.^49^ Purple corn (*Zea mays*) anthocyanin extract containing both nonacylated and acylated anthocyanins fed to *db/db* diabetic mice at a daily dose of 10 or 50 mg/kg body weight for 8 weeks reversed the blood glucose to normal levels and increased muscle GLUT4 gene expressions.^46^ Mulberry nonacylated anthocyanin extract fed to *db/db* diabetic mice for 2 weeks has shown both activated AKT and AMPK and increased GLUT4 level in skeletal muscle.^52^

**Figure 2:**
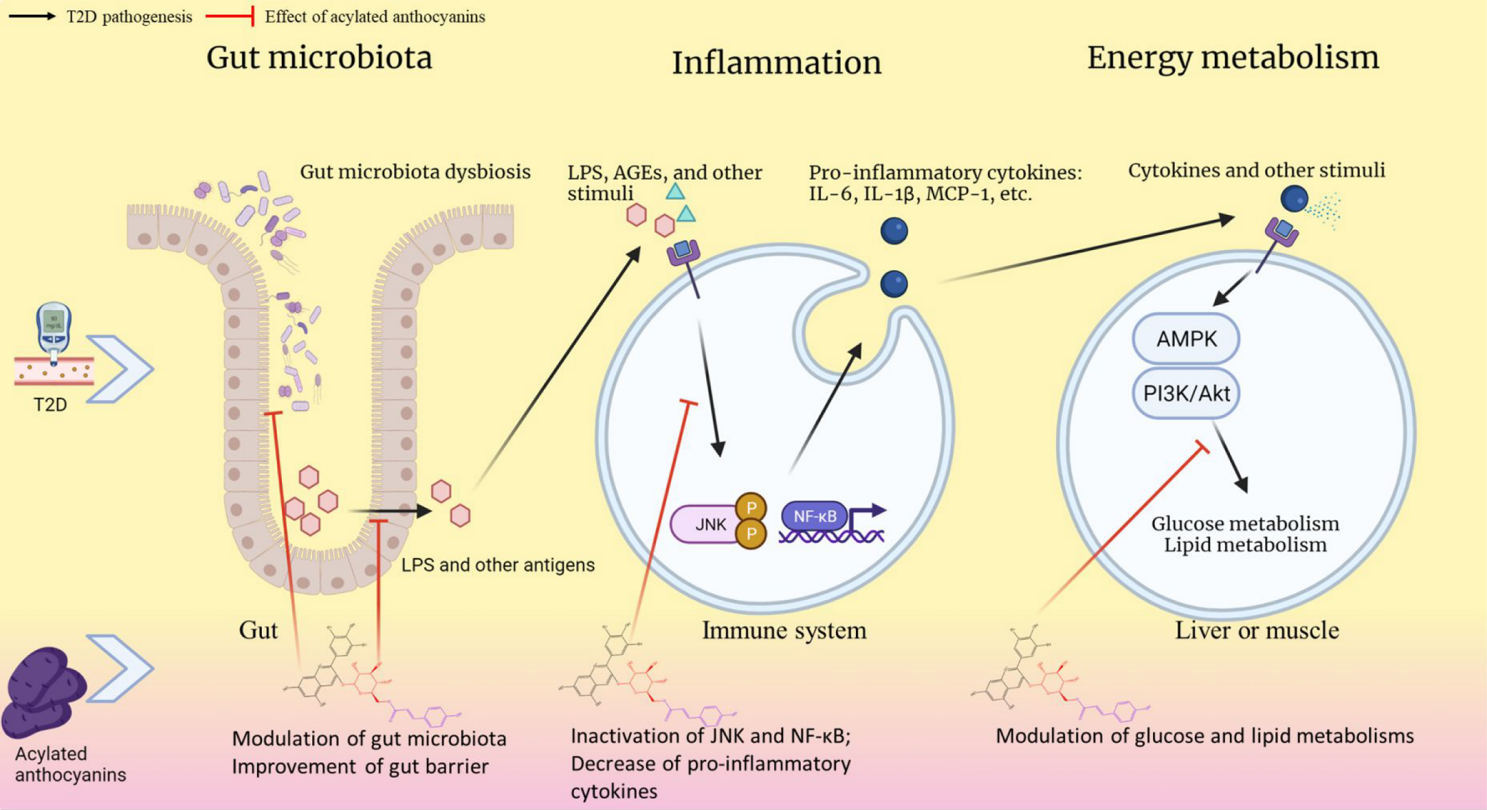
Potential antidiabetic effect of acylated anthocyanins. AGEs, advanced glycation end products; AMPK, AMP-activating protein kinase; IL, interleukin; JNKs, JUN N-terminal kinases; LPS, lipopolysaccharides; NF-κB, nuclear factor-κB; PI3K/AKT, phosphoinositide 3 kinase/protein kinase B; T2D, type 2 diabetes; TNF, tumor necrosis factor.

However, nonacylated and acylated anthocyanins might not always activate AMPK and AKT to the same extent or activate the same downstream effectors. A single oral dosage of an acylated anthocyanin extract from purple carrot and an equivalent dose of two nonacylated anthocyanins (delphinidin-3-*O*-rutinoside and cyanidin-3-*O*-glycoside, 1 mg/kg body weight) have been used to assess their capacities to phosphorylate AKT phosphorylation in the aorta.^54^ As compared to the other two nonacylated anthocyanins, acylated anthocyanin extract has exhibited a higher level of AKT phosphorylation.^54^ This result suggests acylated anthocyanins might play a better role in regulating AKT phosphorylation in T2D (Figure 2). A common downstream target of AKT and AMPK is TBC1D1 which regulates the translocation of GLUT4, lipogenesis, and insulin resistance in T2D.^55,56^ Feeding acylated anthocyanin extract from purple potato (var. “Synkeä Sakari”) at a daily dose of 50 mg/kg body weight for 8 weeks to Zucker diabetic fatty (ZDF) rats decreased hepatic gene expression of the *TBC1D1* and *G6pc* as well as plasma gluconeogenic substrate glycerol; however, nonacylated anthocyanin extract from bilberry did not show similar results.^57^ The purple potato acylated anthocyanins extract, but not the bilberry nonacylated anthocyanins extract, reversed the increased plasma (systemic) glycolysis fluxes in ZDF rats,^58^ while both types of anthocyanins extracts decreased hepatic glycolysis fluxes.^57^ Although a decrease in hepatic glycolysis fluxes by both types of anthocyanin extracts has been observed, the impact of the two types of anthocyanins might have been due to different metabolism pathways since nonacylated anthocyanin extract increased gene expression of hepatic glucokinase, indicating its potential role as a glucokinase activator, while acylated anthocyanin extract decreased the hepatic expression of pyruvate kinase gene (*Pklr* type).^57^ However, contrary results have been reported showing that purple sweet potato acylated anthocyanin extract increased hepatic gene expression of pyruvate kinase (*Pkm* type) and phosphofructokinase (*Pfk* type) in a high-fat diet and STZ-induced diabetic mice.^48^ This difference might be due to the different animal models used in these two studies and/or the different acylated anthocyanin composition in purple potato (var. “Synkeä Sakari”, mainly monoacylated anthocyanins) and purple sweet potato (mainly diacylated anthocyanins).

Other potential different antidiabetic targets between acylated and nonacylated anthocyanins might also play a role in the observed antidiabetic effects due to the structural difference between the two types of anthocyanins. Glycerol-*sn*-3-phosphate acyltransferase (GPAT) can produce phosphatidic acid which is the precursor of triglyceride and glycerophospholipids from acyl-CoA and glycerol-3-phosphate. Suppression of GPAT1 by nonacylated anthocyanins has been frequently observed, for which the potential mechanism might be PPARγ/PKC-mediated.^59,60^ Although both f types of anthocyanins have demonstrated cholesterol- and TAG-lowering effects in animal models and human, acylated anthocyanins have not been proven to work via this route.^4,16,61,62^

### 3.3 Modulation of Inflammation

Insulin resistance in T2D has been attributed to low-grade and chronic inflammation in the liver, skeletal muscles, and adipose tissue.^63^ Thus, preventing inflammation can be an important strategy to manage insulin resistance in diabetes.

This low-grade and chronic inflammation is referred as “para-inflammation”, describing the immune responses where sustained tissue malfunction and stress are induced by a variety of factors, such as reactive oxygen species (ROS), advanced glycation end products (AGEs), and oxidized lipoproteins.^64^ ROS produced by phagocytes can oxidize and degrade lipoproteins to pro-inflammatory products.^65^ AGEs can accumulate under pro-oxidative status (such as aging) and hyperglycemia (such as diabetes).^65^ Para-inflammation may result in maladaptive chronic nonresolving immune activation, inhibition of insulin signaling pathways, and the development of insulin resistance.^65^ Moreover, para-inflammation has been associated with overfeeding or consumption of high-energy food. When caloric intake exceeds the energy expenditure, overloaded tricarboxylic acid cycle intermediates cause an excessive amount of mitochondrial NADH (mtNADH) and ROS. When the excess of mtNADH cannot be handled by oxidative phosphorylation, the mitochondrial proton gradient increases, and electrons are transferred to oxygen, resulting in the formation of free radicals, which might induce inflammation and insulin resistance.^65^ Oxidative stress induced by high caloric intake can activate NF-κB and release of its downstream pro-inflammatory cytokines.^66^ Consumption of a high-fat diet has been reported to lead to the activation of circulating and adipose immune cells *via* Toll-like receptor 4 (TLR4) signaling, causing the subsequent release of pro-inflammatory cytokines.^63^ The inflammation involved in adipose tissue recruitment of pro-inflammatory M1 macrophages, which partly contributes to insulin resistance.^63^

According to a pharmacological review, NF-κB is the main cause of inflammatory-induced insulin resistance in diabetes.^67^ NF-κB, a nuclear transcription factor, is a crucial regulator of inflammatory and immunological responses that could be triggered by a variety of proinflammatory stimuli and oxidative stress, such as cytokines, free radicals, AGEs, and bacterial or viral antigens. The downstream effects include the expression of proinflammatory cytokines (IL-6, TNF-α, IL-1, IL-2, etc.), chemokines (MCP1, IL-8, etc.), immunoreceptors, acute phase proteins, cell adhesion molecules (VCAM-l, vascular cell adhesion molecule-l), growth factors, and inducible enzymes including inducible nitric oxide synthase (iNOS) and cyclooxygenase-2 (COX-2).^67^ The function of NF-κB is controlled by inhibitors of NF-κB (IκB). The deactivated form of NF-κB interacts with IκB proteins in cytoplasm.^67^ Pro-inflammatory signals can activate IκB kinase (IKK) to degrade IκB proteins and release NF-κB p65/p50 heterodimer for nuclear translocation.^68^ Ikk^±^*ob/ob* mice with lower IKKβ expression have been shown to be protected from the development of insulin resistance.^69^ JNKs (JUN N-terminal kinases) are also important to insulin resistance due to their pro-inflammatory role, the JNK pathway is overactive in the muscle, liver, and adipose tissue in T2D, while blocking the JNK pathway has been reported to protect against insulin resistance.^70^

Both acylated anthocyanins and nonacylated anthocyanins have been shown to decrease activation of NF-κB, JNKs and reduce their downstream pro-inflammatory effects in T2D.^71^ Amelioration of inflammation by anthocyanins has suggested improved insulin resistance and glucose metabolism in diabetes.^71^ Oral administration of anthocyanin extracts from purple rice (predominantly cyanidin-3-glucoside) at a daily dose of 700 mg/kg body weight for 4 weeks to STZ-induced diabetic mice has shown decreased levels of blood glucose (ca. 13%), phosphorylated IKKβ, and COX-2 and IL-6 in the heart.^71^ Feeding diets containing 8% wild blueberry (*Vaccinium angustifolium*) powder to ZDF rats for 8 weeks downregulated NF-κB p50, TNF-α, and IL-6 expression in abdominal adipose tissue and liver.^72^ Supplementation of a diet containing 0.2% nonacylated anthocyanin cyanidin-3-*O*-glucoside to high-fat diet-induced and *db/db* diabetic mice for 5 weeks has shown a decreased blood glucose level by 20% and mitigated pro-inflammatory cytokines and insulin resistance, by regulating the JNK pathway.^73^

Supplementation of a daily dose of 700 mg/kg body weight acylated anthocyanin extract from purple sweet potato for 20 weeks has shown improvement of hepatic IRS-1/PI3k/AKT insulin signaling pathway and less activation of JNK1 and IKKβ in high-fat diet-induced obese mice.^45^ Supplementation of daily dose of 700 mg/kg body weight acylated anthocyanin extract from purple sweet potato for 20 weeks decreased blood glucose to the normal level and inhibited the NF-κB p65 nuclear translocation and nucleotide oligomerization domain protein1/2 (NOD1/2) signaling as well as the downstream inflammatory cytokines expression (IL-1β and IL-6, etc.) in high-fat diet-induced diabetic mice.^74^ Feeding acylated anthocyanin extract from black goji berry to mice with atherosclerosis has shown a lower arterial NF-κB p65 and VCAM-1 expression, plasma IL-1β, and TNF-α level, and hepatic CYP7A1 and SREBP-2 level.^75^ Both acylated and nonacylated anthocyanins have been reported to alleviate NF-κB through downregulating TLR4 (Figure 2).^53,71^

Although studies comparing acylated and nonacylated anthocyanins in terms of their ability to inhibit the NF-κB pathway have not been published, one study compared the effects of five structurally different anthocyanins on AGEs formation which can induce the NF-κB pro-inflammatory pathway.^76^ Petunidin-3-rutinoside-(coumaroyl)-5-glucoside, an acylated anthocyanin, was found to have the strongest anti-AGEs formation effects by slowing the glycation progression, followed by diglycosides of anthocyanidins (delphinidin-3-sambubioside and cyanidin-3-sophoroside) and then monoglycoside of anthocyanidins delphinidin-3-glucoside and (pelargonidin-3-glucoside),^76^ demonstrating that acylated anthocyanins may be more effective at suppressing NF-κB activation than nonacylated anthocyanins.

The impact of acylated anthocyanins from different dietary sources on inflammatory pathways may also differ. An *in vitro* study has shown acylated anthocyanin-rich extracts from purple carrots and purple potatoes suppressed lipopolysaccharide (LPS)-induced phosphorylation of JNK and IkBα in mucosal innate immune cells with purple potato anthocyanins extract showing more efficiency.^77^ Another study has compared the effect of acylated anthocyanin extract from purple potato (var. “Synkeä Sakari”) and nonacylated anthocyanin extract from bilberry on hepatic transcriptomic profile in high-fat diet-induced ZDF rats.^57^ Only the potato anthocyanin extract decreased the AP-1 (a transcriptional regulator, activator protein 1) level and IL-1β production, which are downstream effectors of JNK.^57^

Nrf2 is a transcription factor that regulates antioxidant protein production and protects against oxidative damage caused by inflammation or injury.^61^ Nonacylated anthocyanins have been shown to activate Nrf2 in HepG2 cell line and different organs of obese and diabetic animals.^12,61,78^ Acylated anthocyanin fraction of purple sweet potato has not affected Nrf2 activation in Huh-7 cells,^79^ and this effect has not been accessed *in vivo*.

### 3.4 Effect on Gut Microbiota

The human gastrointestinal tract has the largest interface (250–400 m^2^) between the environmental factors and the host. The human gastrointestinal tract harbors at least 10^14^ bacterial cells belonging to over 1000 bacterial species.^80^ The genome of those bacteria has been estimated to include over 3.3 million genes, which is 150 times more compared to the human genome.^80^ The gut microbiota has been reported to have impacts on the digestion, regulation of metabolic pathways, and innate and adaptive immunity.^80^

The dynamics of the gut microbiota profile is influenced by genetic factors, lifestyle, drugs (especially antibiotics), and dietary habits.^81^ Host-symbiotic interactions of the microbiota play important roles in the host physiology. For example, the gut epithelial cells and antimicrobial peptides produced by the gut microbiota contribute to the gut barrier, the first frontline against exogenous molecules and pathogens in the gastrointestinal tract. Gut microbiota influence the gut epithelial morphology, mucus production, secretion of immune factors, and gut permeability when interacting with the epithelial and mucosal immune cells.^82^ Exogenous molecules in the lumen can pass through the damaged gut barrier to the endothelium and even systemic circulation and induce the inflammatory response (Figure 2).^83^ For example, metabolic endotoxemia is linked with the development of obesity and T2D.^83^ In particular, dysregulated gut microbiota composition in obesity and T2D may damage the gut barrier and expose LPS to systemic circulation (para-inflammation), which renders chronic low-grade inflammation linked to insulin resistance, adiposity, and *de novo* synthesis of triglycerides.^83^ LPS can induce the innate immune system response by binding to TLR4 and its coreceptors.^82^ TLR4 belongs to the TLR family, one of the pattern-recognition receptors. When these receptors are activated, myeloid differentiation primary response protein 88 (MyD88, adaptor proteins of Toll-like receptors) is recruited and pro-inflammatory signaling cascades are activated, for example, the aforementioned NF-κB pathway.^84^ Moreover, deletion of MyD88 has been shown to protect against obesity and insulin resistance in mice.^85^

In humans, there are no endogenous esterases to release phenolic acids from the acylated anthocyanins; however, the esterases present in gut microbiota is able to do so.^86^ As aforementioned, due to the lower bioavailability of acylated anthocyanins than nonacylated anthocyanins, more acylated anthocyanins would be exposed to gut microbiota compared to nonacylated anthocyanins.^77^

Gut metabolism of anthocyanins including the absorption, distribution, and excretion were extensively reviewed elsewhere.^16,26^ Briefly, gut bacterial metabolism of anthocyanins involves the cleavage of glycosidic linkages and breakdown of anthocyanidin heterocycle (from C-ring), degradation into phloroglucinol derivatives (from A-ring) and benzoic acids (from B-ring), and O-demethylation, forming simple phenolics and phenolic-sulfated, phenolic-glucuronidated, phenolic-methylated metabolites.^16,26^

The more bioavailable phenolic metabolites may contribute to the biological benefits of anthocyanins mentioned above. For example, protocatechuic acid has been reported to stimulate the insulin signaling pathway and upregulate PPARγ activity, adiponectin release, GLUT4 translocation, and glucose uptake in human adipocytes cell lines^87,88^ and human primary adipocytes cells^89^ as well as have an antihyperglycemic in diabetic rats.^90^

So far, there are no studies investigating the difference in gut metabolites between acylated and nonacylated anthocyanins. However, our previous study has detected novel metabolites of monoacylated anthocyanins from purple potatoes in human urine for the first time, such as hydroxybenzoic and hydroxycinnamic acids as well as glucuronyl and sulfonyl conjugates of protocatechuic acid, of which some might be related to the gut metabolism of acylated anthocyanins.^88,91^ Our results have shown that the absorbed potato acylated anthocyanins are likely to be resistant to deglycosylation since 91% of nonacylated monoglycosylated anthocyanins are deglycosylated after ingestion and form conjugates of aglycones in urine.^91,92^

Despite nonacylated anthocyanins have been frequently reported in reviews^12^ to affect the abundance of fecal *Lactobacillus* spp., *Bifidobacterium* spp., and *Clostridium* spp., contradictory results have also been observed. For example, supplementation of nonacylated anthocyanin extract from blackcurrant (*Ribes nigrum*) has enriched the abundance of *Bifidobacterium* spp. in humans, while anthocyanins from black raspberry (*Rubus racemosus*) has depleted the abundance of *Bifidobacterium* ssp. in rats.^26^ This could be due to different metabolic states, interspecies differences, and structures of anthocyanins. In this review, we examine regulatory effects of anthocyanins on the gut microbiota in the condition of obesity and diabetes for the first time. Table 3 presents a summary of the studies on the effects of anthocyanins on gut microbiota.

**Table 3:** Gut Microbiota Regulation Effects of Anthocyanin in the Conditions of Obesity and Diabetes and the Different Prebiotic Effects between Acylated and Nonacylated Anthocyanins^a^

Source of anthocyanins	Main anthocyanin(s)	Methods	Effect
Anthocyanin extract from black rice (*Oryza sativa* Japonica)^93^	Cya-3,5-diglc, cya-3-glc, cya-3-rut, peo-3-glc	HFD- induced obese C57BL/6J mice; 150 mg/kg bodyweight; 14 weeks	Decreased body weight gain, triglycerides, total cholesterol, steatosis scores and insulin resistance index
			Improved the gene expression profiles involved in lipid metabolism
			Increased the abundances of *Bacteroides*, *Akkermansia*, *Lactobacillus*, *Ruminococcaceae*_*UCG014* and *Alloprevotella* at genus level; at the species level, decreased the proportions of *Dorea_sp._*5–2*Blautia coccoides*, *Lactobacillus gasseri, Mucispirillum schaedleri, Akkermansia muciniphila* and *Parabacteroides merdae*
			Diversity and richness of microbiota were not changed
Freeze-dried strawberry^95^	Not available	*db/db* diabetic mice; diet containing 2.35% freeze-dried strawberry; 10 weeks	α-Diversity indices and β-diversity were different at the phylum and genus levels
			At the phylum level, decreased the abundance of Verrucomicrobia, at the genus level, increased *Bifidobacterium*
			PICRUSt revealed significant differences in 45 predicted metabolic functions
Purified anthocyanins from purple sweet potato (*Ipomoea batatas* Lam.)^99^	Peo-3-sop-5-glc; peo-3-fer- sop-5-glc; peo-3-caf-sop-5-glc; peo-3-caf-hba-sop-5-glc; peo-3-caf-fer-sop-5-glc	Antioxidant activities, proliferative effects on probiotics, and their inhibition on harmful bacteria *in vitro* were tested	Diacylated anthocyanin had the best antioxidant ability and inhibition ability of harmful bacteria (*Staphylococcus aureus* and *Salmonella typhimurium*) followed by monoacylated anthocyanin and then nonacylated anthocyanin
Purified anthocyanin^96^	Cya-3-glc	High fat-high sucrose diet-induced insulin-resistant C57 BL/6J mice; 7.2 mg/kg body weight; 11 weeks	Decreased *Lachnospiraceae* and *Erysipelotrichaceae* and increased the Bacteroidetes/Firmicutes and family Muriculaceae
Purified anthocyanin^97^	Pg-3-glc	*db/db* diabetic mice; 150 mg/kg body weight; 8weeks	Decreased plasma glucose and promoted glucose uptake
			Induced autophagy by modulating Transcriptional factor EB
			Increased abundance of *Prevotella*, Bacteroidetes/Firmicutes ratio, and intestinal barrier integrity
Concord grape freeze-dried powder^33^	Del- and pet- derived acylated anthocyanins	*polygenic obese* C57 BL/6J mice; dose of grape powder normalized to 400 μg/g total anthocyanin content; 12 weeks	Increased abundance of Actinobacteria
Anthocyanins extract from purple sweet potato (*Ipomoea batatas* Lam)^98^	Cya-3-caf-cou-diglc-5-glc, cya-3-fer-sop-5-glc, cya-3-sin-diglc-5-xyl, pg-3-ace-diglc-5-glc, cya-3-hba-oxa-diglc-5-glc, cya-3-diglc-5-glc, cya-3-difer-sop-5-glc, cya-3-caf-fer-sop-5-glc	*In vitro* stimulation of gut microbiota	Induced the growth of *Bifidocterium* spp. and *Lactobacillus* spp. and inhibited the growth of *Prevotella* and *Clostridium histolyticum*
			Increased SCFAs
Anthocyanin extract from black goji berry (*Lycium ruthenicum*)^75^	Pet-3-rut-cou-5-glc as the main anthocyanin	HFD and vitamin-D3- induced atherosclerosis rat, 50–200 mg/kg body weight; 8 weeks	Increased abundance of *Bifidobacterium, Lactobacillus, Roseburia, Akkermansia*, and *Lachnospiraceae_NK4A136_group* and decreased *Prevotellaceae_NK3B31_group*
			Increased gut barrier
Nonacylated anthocyanin extract from bilberry (*Vaccinium myrtillus*) and acylated anthocyanin extract from purple potato (*Solanum tuberosum* var. “Synkeä Sakari”)^37^	Nonacylated anthocyanins dominated by del-3-gal, del-3-glc, cya-3-gal, del-3-ara, cya-3-glc	Zucker diabetic fatty rats; daily doses of 25–50 mg/kg body weight; 8 weeks	Both anthocyanin extracts increased abundance of *Peptostreptococcaceae* sp. and decreased abundance of *Parabacteroides* spp. Acylated anthocyanins decreased *Ruminococcus torques* and *Lachnospiraceae bacterium 4_1_37FAA* abundances
	Acylated anthocyanins dominated by pet-cou-rut-glc and peo-cou-rut-glc		

a Note: Anthocyanidins: cya, cyanidin; del, delphinidin; mv, malvidin; pg, pelargonidin; peo, peonidin; pet, petunidin. Acyl moieties: ace, acetyl; caf, caffeoyl; cou, coumaroyl; fer, feruloyl; hba, hydroxybenzoyl; mal, malonyl; oxa, oxaloyl; sin, sinapoyl; suc, succinyl; pyr, pyranosyl. Sugar moieties: gal, galactoside; rut, rutinoside; sop, sophoroside; xyl, xyloside; glc; glucopyranoside.

Nonacylated anthocyanins extracted from black rice fed to high-fat diet-induced obese mice at a daily dose of 150 mg/kg body weight for 14 weeks did not affect the overall diversity and richness of microbiota but did enrich the abundance of genera *Ruminococcaceae*, *Alloprevotella*, *Lactobacillus*, *Bacteroides*, and *Akkermansia* and species *Blautia coccoides*, *Dorea sp.* 5–2, *Akkermansia muciniphila, Lactobacillus gasseri*, *Mucispirillum schaedleri*, and *Parabacteroides merdae*.^93^ The decreased abundance of *A. muciniphila* has been reported in T2D. Feeding *A. muciniphila* to T2D mice has been shown to improve mucus layer thickness, metabolic function, glucose tolerance, and systemic inflammation.^82^ Pasteurization of *A. muciniphila* also reduced fat mass development, insulin resistance, and dyslipidemia in mice, and the mechanism might be due to a specific protein from the outer membrane of *A. muciniphila* interacting with Toll-like receptor 2 which shapes the host metabolism by regulating bacterial recognition and intestinal homeostasis.^94^ Feeding a diet contaning 2.35% freeze-dried strawberry to *db/db* mice for 10 weeks has been reported to enhance gut microbiota diversity and lower the abundance of Verrucomicrobia.^95^ Supplementation of purified cyanidin-3-glucoside at a daily dose of 7.2 mg/kg body weight for 11 weeks enriched *Erysipelotrichaceae* and *Lachnospiraceae* and increased the Bacteroidetes/Firmicutes ratio and the family *Muriculacea* in high-fat and high-sucrose diet-induced insulin-resistant C57 BL/6J mice.^96^ Feeding a daily dose of 50 mg/kg body weight pelargonidin-3-*O*-glucoside for 8 weeks to *db/db* diabetic mice increased the *Prevotella* abundance and Bacteroidetes/Firmicutes ratio and strengthened gut barrier integrity.^97^ Incubation of potato acylated anthocyanins extract from purple sweet with human gut microbiota enriched the abundance of *Lactobacillus* spp. and *Bifidobacterium*, depleted the abundance of *Clostridium histolyticum* and *Prevotella*, and induced the production of short-chain fatty acids (SCFAs).^98^

Although the prebiotic role of acylated anthocyanins has not been extensively studied, acylation status of anthocyanins might influence the effect of anthocyanins on gut microbiota homeostasis in diabetes. An *in vitro* study assessed the prebiotic activity and antioxidant ability of purified diacylated anthocyanin, monoacylated anthocyanin, and nonacylated anthocyanin, showing diacylated anthocyanin has the most potent antioxidant ability and inhibition on the growth of harmful bacteria (*Salmonella typhimurium* and *Staphylococcus aureus*) followed by monoacylated anthocyanin and then nonacylated anthocyanin.^99^ Obese mice fed with Concord grape rich in acylated anthocyanin for 12 weeks showed the highest level of Actinobacteria compared to supplementation of berries rich in nonacylated anthocyanin, followed by obese models.^33^*Peptostreptococcaceae* has been shown to be significantly lower in T2D patients,^100^ and both nonacylated anthocyanin extract from bilberry and acylated anthocyanin extract from purple potato increased the abundance of *Peptostreptococcaceae* in ZDF ras.^37^ In addition, only the acylated anthocyanin extract from purple potato enriched the abundance of *Ruminococcus torques*, *Parabacteroides disdasonis*, and *Lanchnospiraceae bacterium 4_1_37FAA* in ZDF rats.^37^*P. distasonis* is a dominant species producing propionate in human gut flora, contributing to the increased level of propionate in the rats fed with acylated anthocyanin extract.^37^*Parabacteroides* has been shown to have a positive correlation with serum proinflammatory cytokine IL-17 and splenic CD4+ Th17 cells in arthritic mice^101^ and to be more enriched in patients with nonalcoholic steatohepatitis.^102^*R. torques* is able to decrease the gut barrier integrity and has been shown to have a positive correlation with insulin resistance in obesity.^103^ As for gut metabolites, only acylated anthocyanin extract in contrast to nonacylated anthocyanin increased cecal SCFAs and succinate levels in diabetes,^37^ and a study showed that cecal succinate is a substrate for intestinal gluconeogenesis and benefit glycemic responses and hepatic glucose production.^104^ However, the gut microbiota-modulating effect of acylated anthocyanins deserves further study in different models as well as different physiological and pathological conditions (Figure 2).

## 4 Conclusion and Future Perspectives

Anthocyanins have been reported to affect energy metabolism, inflammation, and gut microbiota in T2D. The physicochemical properties, bioavailability, and metabolism differ between acylated and nonacylated anthocyanins. Acylated anthocyanins with higher stability can pass through the upper gastrointestinal tract and reach the colon, where they are extensively metabolized by gut microbiota. Glucose transporters are involved in anthocyanin absorption, and different glucose transporters are responsible for the absorption of acylated and nonacylated anthocyanins. There are indications that acylated and nonacylated anthocyanins might have different effects on T2D. Acylated anthocyanins have a greater inhibitory effect on α-glucosidase and α-amylase compared to nonacylated anthocyanins. Acylated and nonacylated anthocyanins modulate key enzymes or metabolites involved in energy metabolism and inflammation to different degrees; for example, acylated anthocyanins induce a higher level of AKT phosphorylation and AP-1 activation. Acylated anthocyanin has a better antioxidant ability and inhibition on the AGEs formation and growth of harmful bacteria compared to nonacylated anthocyanins. Acylated anthocyanins can improve the gut barrier and microbiota composition, suppress the pro-inflammatory pathways, and modulate glucose and lipid metabolisms. Drawing clear conclusions about the different biological activity in diabetes between acylated and nonacylated anthocyanins based on the current literature is challenging, as the studies differ in design and analytical methods, and most importantly the insufficient data from comparative *in vivo* study between two types of anthocyanins. Further studies are needed to compare the effects of structurally different anthocyanins. The currently available evidence suggests that acylated anthocyanins may have greater potential modulation effects on energy metabolism, inflammation, and gut microbiota in T2D compared to nonacylated anthocyanins.

## Abbreviations

ACC: acetyl-coenzyme A carboxylase
AGEs: advanced glycation end products
AKT: protein kinase B
AMPK: adenosine monophosphate-activated protein kinase
AP-1: activate protein 1
G6pase: glucose 6-phosphatase
GLUT: glucose transporter
GPAT: glycerol-*sn*-3-phosphate acyltransferase
HbA1c: glycated hemoglobin
HFD: high-fat diet
IL: interleukin
IRS-1: insulin receptor substrate 1
LPS: lipopolysaccharide
PEPCK: phosphoenolpyruvate carboxykinase
PI3K: phosphoinositide 3 kinase
MCP-1: monocyte chemoattractant protein-1
NF-κB: nuclear factor κB
Nrf2: nuclear factor erythroid 2-related factor 2
PPAR: peroxisome-proliferator-activated receptor
ROS: reactive oxygen species
SGLT1: sodium-glucose cotransporter 1
SOD: superoxide dismutase
SCFAs: short-chain fatty acids
TNF-α: tumor necrosis factor-α
